# Stakeholder Narratives on Trypanosomiasis, Their Effect on Policy and the Scope for One Health

**DOI:** 10.1371/journal.pntd.0004241

**Published:** 2015-12-11

**Authors:** Catherine Grant, Neil Anderson, Noreen Machila

**Affiliations:** 1 Institute of Development Studies, Falmer, Brighton, United Kingdom; 2 The Royal (Dick) School of Veterinary Studies, The University of Edinburgh, Roslin, United Kingdom; 3 School of Veterinary Medicine, University of Zambia, Lusaka, Zambia; Foundation for Innovative New Diagnostics (FIND), SWITZERLAND

## Abstract

**Background:**

This paper explores the framings of trypanosomiasis, a widespread and potentially fatal zoonotic disease transmitted by tsetse flies (*Glossina* species) affecting both humans and livestock. This is a country case study focusing on the political economy of knowledge in Zambia. It is a pertinent time to examine this issue as human population growth and other factors have led to migration into tsetse-inhabited areas with little historical influence from livestock. Disease transmission in new human-wildlife interfaces such as these is a greater risk, and opinions on the best way to manage this are deeply divided.

**Methods:**

A qualitative case study method was used to examine the narratives on trypanosomiasis in the Zambian policy context through a series of key informant interviews. Interviewees included key actors from international organisations, research organisations and local activists from a variety of perspectives acknowledging the need to explore the relationships between the human, animal and environmental sectors.

**Principal Findings:**

Diverse framings are held by key actors looking from, variously, the perspectives of wildlife and environmental protection, agricultural development, poverty alleviation, and veterinary and public health. From these viewpoints, four narratives about trypanosomiasis policy were identified, focused around four different beliefs: that trypanosomiasis is protecting the environment, is causing poverty, is not a major problem, and finally, that it is a Zambian rather than international issue to contend with. Within these narratives there are also conflicting views on the best control methods to use and different reasoning behind the pathways of response. These are based on apparently incompatible priorities of people, land, animals, the economy and the environment. The extent to which a One Health approach has been embraced and the potential usefulness of this as a way of reconciling the aims of these framings and narratives is considered throughout the paper.

**Conclusions/Significance:**

While there has historically been a lack of One Health working in this context, the complex, interacting factors that impact the disease show the need for cross-sector, interdisciplinary decision making to stop rival narratives leading to competing actions. Additional recommendations include implementing: surveillance to assess under-reporting of disease and consequential under-estimation of disease risk; evidence-based decision making; increased and structurally managed funding across countries; and focus on interactions between disease drivers, disease incidence at the community level, and poverty and equity impacts.

## Author Summary

This paper explores the differing opinions of various stakeholders in relation to trypanosomiasis, a widespread and potentially fatal disease spread by tsetse flies which affects both humans and animals. It is an important time to examine this issue as human population growth and other factors have led to migration into new areas which are populated by tsetse flies, and this may affect disease levels. This means that there is a greater risk to people and their livestock, and opinions on the best way to manage the disease are deeply divided. Cross sector working hasn’t been used historically, but breaking down the barriers between social scientists, natural scientists and the expertise of the community could lead to better disease management.

## Introduction

### Trypanosomiasis

Trypanosomiasis is caused by protozoan parasites transmitted by the tsetse fly [1]. In people, the parasites cause Human African Trypanosomiasis (HAT, also known as sleeping sickness), a serious illness which kills without appropriate treatment. From a peak of approximately 300,000 cases per year in 1995, cases have declined to less than 10,000 per year as a result of improved control measures [2]. However, an estimated 70 million people in Africa live in areas of risk for HAT. The disease principally affects remote rural communities in areas with poor health infrastructures and inequitable access to health care [3]. Under-reporting of cases is a significant problem as it is for all the neglected tropical diseases and the actual number of cases is greater than that reported [4]. In livestock, the disease causes African Animal Trypanosomiasis (known locally as *nagana*) and causes severe production losses.

### Situation in Zambia

Trypanosomiasis cycles naturally in wildlife populations which form a reservoir of infection with sporadic spill-over into human populations [5]. Only the acute form of HAT, caused by *T*. *b*. *rhodesiense* is found in Zambia [6]. It is widespread in the Eastern and Northern provinces with 3.5% of the total national population assessed to be at varying risk of infection [2]. Ecological factors such as climate, rainfall and vegetation in these areas support tsetse vector (*Glossina* species) populations and a sylvatic cycle in wildlife [7]. Zambia has a vast area of wildlife, National Parks protect 6.4%, Game Management Areas (GMAs) cover 15.6% and forest reserves cover 7.2%, so 29.2% of the country is protected in theory [8]. The state policy of creating national parks has been claimed by some to have supported an expansion of tsetse populations [7]. GMAs differ from national parks in that they are zoned for wildlife utilisation, mainly through commercial safari hunting, and also allow human residency, and there is an expanding human population in many and habitat loss occurs alongside this [9].

The risk of trypanosomiasis has been increased by human population growth and migration of people who bring their livestock into tsetse-inhabited areas with little historical influence from humans due to low human population densities. Disease transmission in new human-wildlife interfaces such as these is a greater risk and opinions on the best way to manage this are deeply divided [10]. Many anthropogenic activities reduce tsetse populations due to their sensitivity to ecological change, the extent of which is dependent on the degree of habitat degradation. Similarly, wildlife habitat may also be reduced as wildlife populations retreat due to human activity. Nevertheless, this transition period is particularly risky as the flies are still prevalent and opinions on the best course of action are extremely divided. Given the wide range of potential host species for trypanosomes, ecosystems within the tsetse fly belts are likely to continue to sustain trypanosomiasis indefinitely.

In Southern Africa conservationists have historically considered the presence of tsetse, and thus trypanosomiasis, as a factor protecting natural areas which would otherwise be invaded by people and livestock [11]. As such, they have supported policy decisions based on inaction around disease eradication [12]. However, development professionals have perceived tsetse as preventing the expansion of productive agriculture and thus an obstacle to poverty alleviation and they have prioritised its control. Efforts to control trypanosomiasis have varied in intensity since they became institutionalised in the colonial era. During that time, trypanosomiasis control was a priority issue and around a quarter of the colonial research budget was focused on sleeping sickness control through major treatment campaigns for people, or wider efforts to push back the fly belts [13].

### Trypansomiasis and One Health

The need for a One Health approach was apparent even then, as the differing approaches of the French and British showed a lack of interdisciplinary thinking, with the British focused on tsetse and the French on medical issues [12]. More recently, the introduction of the Structural Adjustment Programme (SAP) by the International Monetary Funds (IMF) meant that animal disease control programmes which had predominantly been managed by the government, were transferred to the private sector in the 1980s/1990s in an effort to reduce the public administration role in veterinary services management [14]. Since this time, control methods focused on specialised technological solutions rather than more holistic One Health approaches and this polarised conservationists and veterinarians/agriculturalists resulting in conflict and disagreement in policy making.

One Health emerged during the early 21st century, when zoonotic disease outbreaks created numerous international crises [15]. Scientists and governments realised more interdisciplinary collaboration was needed to prevent and control zoonoses, and that ‘such collaboration should include not only physicians and veterinarians, but also wildlife specialists, environmentalists, anthropologists, economists and sociologists, among others’ [16]. The term ‘One Health’ was suggested as a concept to nurture this interdisciplinary collaboration [16].

### Aims of the paper

The focus, support and funding for trypanosomiasis has changed over the years, but not necessarily for scientific reasons. This paper focuses on exploring the following:

- The narratives taken by different actors in the field
- Why different narratives have become dominant at different points in history.
- The power relations of stakeholders.

This is reflected on from a One Health perspective, with opportunities for interdisciplinary working—missed as well as taken—considered. This is a crucial area of reflection as disease narratives shape disease policy, with consequences for disease eradication and resulting impact on livelihoods, health and poverty. The flow of historical, political, social and economic knowledge and policy have influenced the management of trypanosomiasis; and this study, focusing on Zambia, adds to the evidence base on knowledge flows between different stakeholders across time. The resulting observations and recommendations will potentially increase our understanding of trypanosomiasis thinking and policy, as well as how One Health concepts can be applied, which then may be able to be extended to further national contexts.

## Materials and Methods

### Ethics statement

ERES Converge IRB, 33 Joseph Mwilwa Road, Rhodespark, Lusaka, Zambia provided ethics approval and approval documents have been submitted.

### Method

A qualitative case study method was used to examine the narratives on trypanosomiasis in the Zambian policy context through a series of key informant interviews conducted from July to October 2013. Interviewees were selected to represent a broad range of perspectives. They included officials from international organisations, researchers (from public and private sectors) and local activists from a variety of perspectives acknowledging the need to explore the relationships between the human, animal and environmental sectors. The choice of stakeholders was based on a literature review of trypanosomiasis policy and its history in Zambia alongside an internet search using key words. The identification process included reviewing donors and international bodies working in the area and searching their websites, a wider search of relevant actors and consultation with experts in the field with experience of working in this area. Key topic areas to discuss and potential questions to explore were also noted during this process. Stakeholders identified were then categorised under the following headings and these people were contacted for face to face interviews, or if this was not possible, interviews over the phone.

- Wildlife departments
- Conservation NGOs
- Veterinary and tsetse control departments
- Ministries of agriculture
- Ministries of health
- Cotton companies and unions
- Tourism operators
- Private wildlife operators and hunting concessions
- Local government
- Intl Atomic Energy authority projects re sterile male tsetse control; pan-African trypanosomiasis control system linked to IBAR
- EU, DFID, FAO tsetse control programmes
- GALVMED/ILRI nexus (re vaccine development)
- WHO and HAT mapping
- Media– especially around human trypanosomiasis outbreaks and seasonal peaks or tourists becoming ill

These were then anonymised into the categories into Table 1 to allow to allow actors to speak freely about their opinions and views without worry of these being published. In each area we interviewed a range of actors using the question themes outlined in Table 2. These included those from veterinary, tsetse control, wildlife, public health and medical backgrounds. This sample may not be large enough to provide a definitive picture but part of the reason for conducting this research is to expose individual biases within sectors and analyse how these biases may affect policymaking. The box of question themes provides details of the areas we covered in the interviews and these actors were asked to give their opinion on the policy context, with our results being extracted from coding and analysing these in depth interviews. Verbal consent was obtained prior to commencing all interviews, with the resulting transcripts entered into Microsoft Word and coded manually. Examining the narratives within specific social and institutional frameworks allowed for critical analysis of the policy processes involved.

**Table 1:** Information on stakeholder groups interviewed.

Interviewee Domain	Number of Respondents
**International Organisations**	**4**
**Private Sector**	**2**
**Charities**	**3**
**Government**	**4**
**Unions**	**1**
**Academia**	**6**
**Total**	**20**

**Table 2:** Question themes.

Question themes
• Personal context
• History of trypanosomiasis
• Describe the current debate on trypanosomiasis. How is the focal disease understood, labelled, differentiated (or not), prioritised or neglected as part of a cluster of diseases/health issues?
• Describe the impact of trypanosomiasis on people, animals and the environment
• Which drivers of disease are seen as most significant?
• What control options do you favour and why?
• Policy processes/narratives: What are the key actor networks? Who do you work with and who don’t you? Who should lead the response? How are policy priorities formed? How has land use changed? Is this important? Who has influenced this? Who influences policy? What are the drivers?
• Do people working on land use work with people working on disease control?
• Does ecosystem change affect disease? What kinds of ecosystem change are seen as significant, and which are ignored?
• With limited funding, should trypanosomiasis be a priority? Why?
• Is trypanosomiasis going up or down the policy agenda? What are the drivers?
• Do people collaborate? Is this useful? (One Health)
• What kinds of spillover and transmission dynamics are identified as important?
• Which groups of people are identified as vulnerable and why?
• What poverty impacts are identified?

The review of the literature on trypanosomiasis policy and its history in Zambia was conducted and analysed in relation to the interview content. The information gathered from the literature review was included in the results alongside the interviews and it was also used to find the interviewees and formulate the questions.

## Results

### Narratives identified

**Fig 1:**
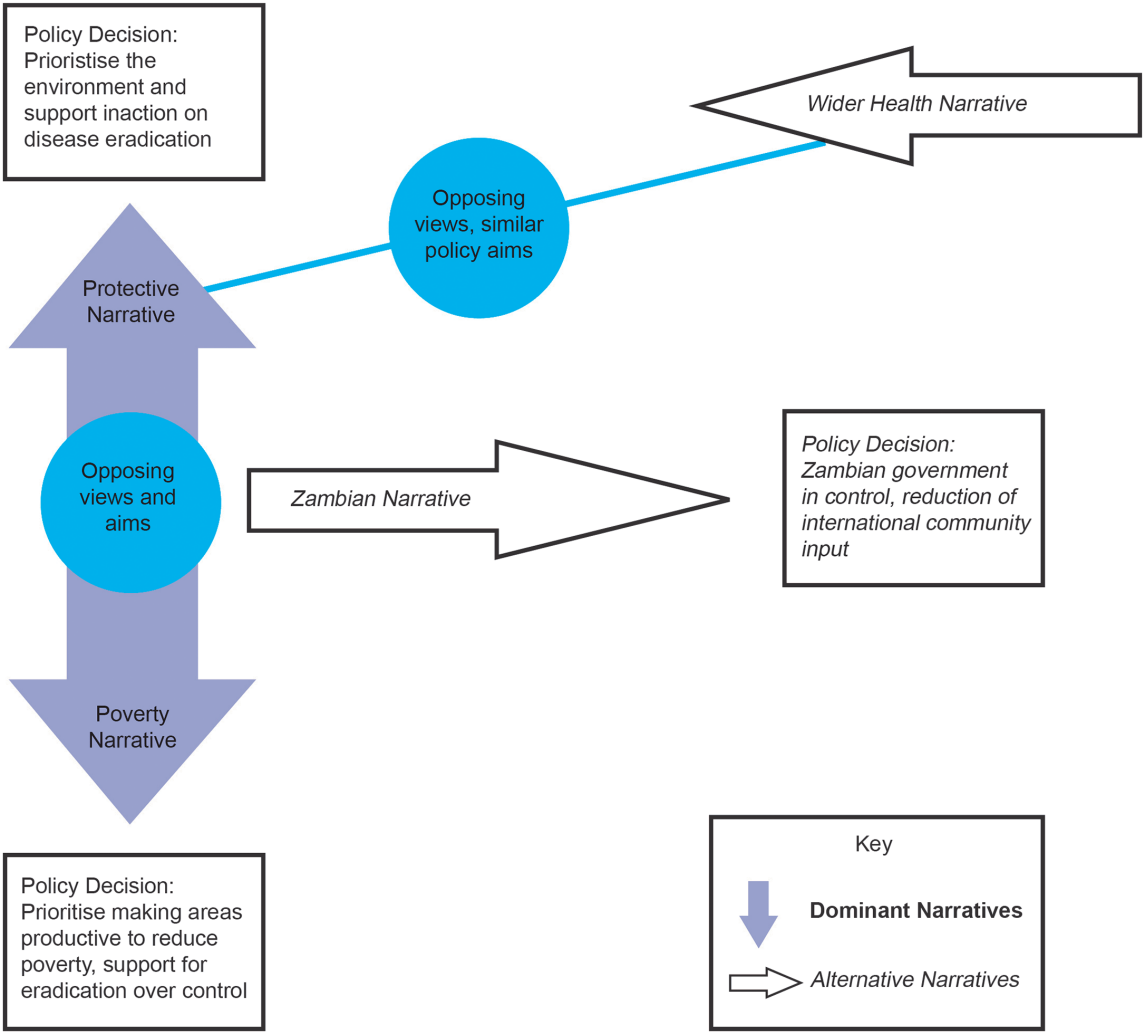
Diagram showing the four narratives.

Two dominant narratives and two alternative narratives have emerged regarding trypanosomiasis in Zambia. These are outlined in Table 3 and Fig 1. The first dominant narrative is the ‘protection narrative’ which maintains that the disease is protecting the national parks, surrounding GMAs and gazetted forest reserves from being invaded by people and livestock, and that policy decisions which prioritise the environment, but which support inaction in relation to eradicating the disease are seen as important (e.g. preventing the effects of deforestation for charcoal, poaching and killing of wildlife and destruction of the natural environment). Interviewees working in the professional hunting industry, environmentalists and environmental activists supported this protection narrative. They believe that ‘tsetse are keeping the area natural and wild’. One interviewee stated:

*Tsetse are still the biggest obstacle to wild areas being taken over by farmers and cattle*. *Tsetse protect the environment and stop farmers encroaching on land so I don’t want tsetse control*. *Farmers move into areas and then hit the tsetse zone and cattle die*. *This protects the land*. *The flies move though*, *so this affects the way things operate*.

(Interviewee)

**Table 3:** Summary of the four policy narratives.

Narrative	Actors who support this	Summary	Policy they support
Protection	Professional hunting industry, environmentalists and environmental activists	Tsetse protect the environment from anthropogenic change	Prioritize the environment; inaction over disease control
Poverty	Tsetse control and ecology section, livestock sector NGO and also the cotton industry	Tsetse is a threat to rural livelihoods	Prioritize making areas productive to reduce poverty
Wider Health	Medical and public health actors	More prevalent diseases are a priority	Inaction over disease control
Zambian	International community, Zambian activists	Zambian government ‘ownership’ of policy	Zambian government in control, reduction of international input

Secondly, the ‘poverty narrative’ maintains that trypanosomiasis is causing poverty in areas which could be made productive and that the parks, GMAs and forest areas must be cleared of trypanosomiasis to protect the livelihoods of the people and allow poverty alleviation. The resulting policy aim is for eradication over control. However, a lack of focus and funding for trypanosomiasis was identified as a major barrier to progress. The tsetse control and ecology section, livestock sector NGO and also the cotton industry, in particular focused on this narrative. They believe that it is in the economic interests of farmers to expand the areas within Zambia that can be used for animal rearing and they want to help this process as it will be beneficial for people.

*People have to move away into marginal and unproductive areas*. *This means that poverty increases and livestock numbers go down*. *This is a vicious circle and poverty will go up if people cannot own livestock*. *Livestock is very critical for livelihoods*. *Without them farmers will be poverty-stricken and dependent on the Government*.

(Interviewee)

There is a key tension between the two narratives because an ecosystem of human settlement, cultivation and livestock keeping opposes the wildlife, woodland savannah and tsetse ecosystem that sustains trypanosomiasis occurrence [17]. Competing ideologies will push for differing policies, which will have a vastly differing impact on tsetse, human and livestock populations, thereby affecting disease levels.

Two alternative views have also been identified. The ‘Zambian narrative’ maintains that the international community had put a lot of emphasis and effort into eradicating trypanosomiasis in the past, but now responsibility for this has been passed over to the Zambian government as constituted, which should take full control and put in place measures to deal with the problem. This has provided international agencies with a justification for withdrawing funding, without the gap necessarily being filled by the Government.

*‘Zambians need to develop policies and know what is best for Zambia’*

(Interviewee).

The final narrative identified is the ‘wider health narrative’, which maintains that Zambia has multiple health and environmental issues and that funding should be directed towards other more important health issues. Furthermore, within this narrative, the public sector prioritises livestock diseases of trade for control before considering those with greater direct impact on health in local communities.

*'The main health problems are HIV and malaria*. *Trypanosomiasis isn’t on the radar*. *The Zambian Government do not have the resources to keep areas tsetse free and we should focus on more prevalent diseases*.*'*

(Interviewee)

Within these narratives there are also conflicting views on the best control methods to use and different reasoning behind the pathways of response. Diverse actors and stakeholders have held differing opinions over time and the power of these actors has affected various policies influencing both the strategies for dealing with the disease and the very dynamics of the disease itself. Some policies, such as those aiming to protect the environment, wildlife or change land use, have had unintended consequences on factors influencing tsetse populations (host availability, climate, and vegetation), or on the interaction between people, livestock and tsetse, thus affecting disease levels [18].

### Focus on African Animal Trypanosomiasis

International organisations, the cotton sector and most other stakeholders focused on animal trypanosomiasis rather than human trypanosomiasis, and so the focus of this paper is often on AAT rather than HAT. The main emphasis from the interviewees' responses was the disease’s economic impact on the country and the agricultural sector. This single focus seemed prevalent, rather than a holistic take on the issue. The tsetse control and ecology section of Government stated: 'The major emphasis is on cattle, this is the biggest threat. Most cases of human trypanosomiasis are not confirmed. The Ministry of Health deals with these, we don’t look into this. Our big focus is that tsetse-free areas stay tsetse free.' (Interviewee). In terms of One Health working, this seemed limited to occasional contact. 'We collaborate with the Ministry of Heath but our role is limited to animals.’ (Interviewee)

### Trypanosomiasis control

These differing views on the importance of trypanosomiasis mean there has been, and remains, great controversy about how to control the disease. Methods and views depend greatly on the narrative and perspective of stakeholders. The scientific community has debated these issues since tsetse and trypanosomiasis control became institutionalised in colonial times, but the validity of these debates is often not what drives policy and programming [12].

The history of trypanosomiasis and other diseases goes some way to explain policy and decision making in relation to control options, and also shows the grave effects control options can have on the population. Elimination of wildlife was once seen as a favourable policy due to the ‘Great Rinderpest Epizootic’, which reached Zambia in 1892 and contributed to the wildlife population decline and a significant reduction in tsetse distribution, bordering on eradication from extensive areas [10]. Eastern Zambia was mainly clear of tsetse, making rearing cattle more viable in the Luangwa valley near the end of the nineteenth century. Around this time, from 1897, the first game reserves were set up by the British to, ‘save Africa’s game,’ [19]. This greatly affected poverty levels, as laws preventing the ‘hunting and trapping of animals and forbidding the sale of gunpowder to Africans’ were made at the same time as their cattle had been killed by disease or seized, cutting off livelihood options and causing poverty [19]. This illustrates the economic and community benefits gained from wildlife.

Despite these outbreaks, small, relic populations of tsetse survived and spread into suitable areas once hosts became available due to the quick repopulation of wildlife. This led to an escalation of the tsetse density in the Luangwa valley, which then expanded ‘southward and eastward onto the plateau areas, resulting in new outbreaks of bovine trypanosomiasis’ [7]. An example of how policy is made based upon the experience of recent history and also showing the competing aims of different groups depending on whether their focus is disease control or wildlife protection, the Government of Northern Rhodesia blamed the wildlife movement for this outbreak, and in 1942 they created the Department of Game and Tsetse Control. The main aim assigned to them was to eliminate tsetse wildlife hosts as a control strategy[20, 21]. There was public opposition to this strategy and it was abolished in 1960 [7].

Wildlife groups still criticise what has been done in the past to control tsetse and trypanosomiasis (interviewee). For example the policy of introducing game fences following the recovery of game and tsetse fly, after the previously described outbreaks which left these areas under threat. One interviewee referred to this policy as ineffective, stating that there were, 'game and cattle fences where[…]a hunter [was] stationed every mile to unnecessarily shoot everything that came over the line’, (interviewee). There was also some anger at a failure to use evidence available at the time showing that not all wildlife species were fed on by the tsetse.

Barriers were also introduced in order to contain tsetse flies, and efforts were made to reverse the reinvasion [22]. However, it has been argued that 'tsetse barriers only have a social function…and they are also a reference point for people, but there is no point of them for tsetse flies. Fly gates are completely useless and are only there so people can show that they are doing something.' (Interviewee)

Despite this, fly gates still nominally exist in Zambia today, with one interviewee stating that he had stopped a guard at a gate and was told they had only found one tsetse fly this year. Research has also acknowledged the ‘technical difficulties and expense associated with the construction and maintenance of the barriers which were not totally effective’ due to the subsequent reinvasion of tsetse in the reclaimed ‘tsetse-free’ areas [22]. ‘The advent of new control technologies subsequently led to a reappraisal of tactics’ [22].

By 1968, spraying using endosulfan began. Odour-baited targets were instituted in 1970 to reduce areas infested by tsetse [7]. Together, game fences, aerial spraying, bush burning, and use of odour-baited targets all aimed to ‘reduce the expansion of tsetse further away from the Luangwa and Zambezi Valley’ ecosystems. These control strategies were limited to communal areas, while in the national parks wildlife and tsetse were left to interact without interference [7].

During the interviews we ascertained current views on control. The Tsetse Section, coming from a poverty narrative believe that eradication of the tsetse fly is key and that, although there are effective methods to do this, it is impossible with the financing available. They believe that,

The best method for dealing with tsetse is aerial spraying–this is very expensive but very effective. Targets work quite well but they only suppress the fly. When you go for control you go back to where you started from. We need to eradicate the fly, so we can clear areas and do surveillance. We really want to start aerial spraying in the next few years. We want to do 5,000 sq km to 10,000 sq km annually. We just need to get the funding to do this.

(Interviewee)

They believe it is necessary to utilise tsetse free areas because keeping them productive for livestock rearing and farming helps to stop the fly thriving, 'if an area is eradicated from flies, we must use it to ensure it stays tsetse free' (Interviewee). The Tsetse Section of the Government Veterinary Department has a strategic plan up until 2017 but not the funding to implement it. Most other interviewees also agree this is the most effective method, but warned that, 'if you leave any pockets of tsetse, you would experience reinfestation’ (Interviewee). International organisations mentioned that they support the use of insecticide-treated targets with nets to clear the flies and ensure no reinvasion. This can reduce the tsetse population to one per cent and chemotherapy reduces trypanosomiasis in animals.

Everyone interviewed stated that at the moment it is only the Government trying to clear tsetse, no outside organisations are working in this area. Many were critical of this Zambian narrative emphasis and felt that more funding was needed. 'Another issue is that retired professionals have not been replaced. These were people that were highly regarded. There is a big human-resource issue in trypanosomiasis control' (Interviewee). The only Government motivation for control is to clear the tsetse so they can use the area for settlement and for cattle. It was suggested that, ‘Population growth is one of the main drivers—if not the driver—of what’s going on’ (Interviewee).

If land use is changed, for example by clearing forested areas to provide land for cotton growing as sanctioned by the Government, it can be a control measure in itself as it disrupts the ecosystem needed to provide a habitat for the tsetse. The cotton sector emphasised that:

There have been changes to the environment. Cotton growing has increased. This means that vegetation that supports the fly and where it lives goes away and is replaced by farmland and this pushes the fly away. There is a link between an increase in farming and fly reduction. With new settlements, the fly is pushed further away from people. There is less trypanosomiasis now because the flies have gone.

(Interviewee)

This conflicts with the prevalent view that national park policies have resulted in an expanding fly belt. It also conflicts with the view from tsetse experts that tsetse flies themselves adapt to new environments, implying that their number can still increase in the future after the initial reduction from cleared land.

There were further critics of the Zambian narrative, who explained that, 'projects can keep systems going but when they leave everything stops working. The history of livestock development is better when there is external help.' (Interviewee). Another criticism of government management was that, ‘in Zambia they do big schemes and if they don’t work then they just carry on. There is no evaluation' (Interviewee). However, those advocating the Zambian narrative emphasised the need to look at how disease control priorities affect communities. As an example:

They used the national parks legislation which came at the same time as tsetse control. Both came from outsiders and had colonial influence. Neither is Zambian. Hunters wanted to protect the land and their businesses and this is why the national parks were made. It is useless for outsiders to come in and say, don’t use the trees. Local people know the importance of not depleting their resources and they have the knowledge to protect them and not overuse. Outsiders do not have this knowledge. Zambians need to develop policies and know what is best for Zambia.

(Interviewee)

Those coming from a protection narrative seek different control options to protect the land, therefore potentially keeping the tsetse population high. Preserving natural resources is important, but these wild areas could serve as a reservoir of wildlife trypanosomiasis, due to the fact that the main necessities needed for tsetse survival are appropriate hosts, climate and vegetation [23]. Current conservation strategies, including poaching reduction to increase wildlife populations, favour enrichment of the wildlife/woodland savannah/tsetse ecosystems that sustain the perseverance of the disease.

### Funding for trypanosomiasis research and control

Funding issues are looked upon differently by each of the different narratives. This has a major impact on how much work can be done, and what kind of interventions are possible. The international organisations, generally looking from the Zambian narrative, believe that the Zambian Government should have the power and responsibility to decide how to use funding and that international organisations are simply there to support what the Government wants. However, with this power comes the responsibility for decision making and finding funding for control of diseases, including trypanosomiasis. This seems in part to be a justification for withdrawing funding and the gap does not seem to have been filled by the Government.

From 1986 donor assistance towards control was directed through the Regional Tsetse and Trypanosomiasis Control Programme (RTTCP) [23]. Its initial objective was to eradicate tsetse from the common fly belt [24]. However, ‘area-wide’ control was not successful so the focus was changed to smaller-scale community-based projects [23]. Soon after this all activities began to decline and eventually closed down [23]. This seems to be partly due to restricted finances and a belief in a need to focus on the wider health narrative, as well as international donors passing over the responsibility to the Zambian Government.

This was confirmed by the Tsetse Section, which explained that in the past they relied on donor funding but the projects have all come to an end.

By 1992 a lot had been achieved but funding ran out and the project was handed over to the Government. We try to target the vector with very little funding. It is hard to undertake sustainable tsetse control under these conditions and the Government can’t fund programmes adequately … This means that areas where the fly was eradicated have become (re)populated…. We know of better control methods but we can’t afford to use them.

(Interviewee)

This lack of government funding causes frustration to those within the ministries in charge of dealing with trypanosomiasis as, considering the issue from the poverty narrative, they believe trypanosomiasis affects livelihoods and causes poverty. They believe funding issues to be a major problem and explain that it is a challenge to provide services to farmers, where a quarter of the animals have trypanosomiasis. Additionally, only 30 per cent of the budget they asked for was approved, and they only received 10 per cent of this (Interviewee). This affects the control options available to them and they cannot meet their obligations for control, instead they have to rely on surveillance. There are a few other options for funding and the Pan African Tsetse and Trypanosomiasis Eradication Campaign (PATTEC) is active in Zambia. There is a Zambian PATTEC coordinator whose work is captured under the Government’s national strategic plan, and is also trying to obtain funding from the African Development Bank.

Another issue brought up by interviewees was that government funding is now on a regional basis. Funding is decentralised down to the lowest level. This has resulted in spreading of meagre financial resources with little impact on tsetse and trypanosomiasis control. As mentioned above, the local political structures are not effective at managing programmes and enforcing regulations, with important disease, ecological and poverty effects for the rural population. As stated by an interviewee of the tsetse control section: ‘This makes implementing tsetse control programmes difficult as you are at the mercy of regional decision making; it makes it very difficult to make an impact'.

Challenging the perspective that more funding is needed for trypanosomiasis is the wider health narrative. This narrative argues that Zambia has multiple health issues, under-reporting of trypanosomiasis may be a contributing factor to this viewpoint. People from this narrative do not feel that the available limited funding should be focused on this area. Government priorities should be on more prevalent diseases, such as HIV/AIDS and malaria, and increasingly people focus on cancer and lifestyle diseases such as diabetes and high blood pressure. During the interviews it was mentioned that the diseases focused on,

[…]are not based on research but on the issues important to those in power. For example, when the president’s son died of AIDS, it was talked about more and there was lots of donor funding…The donors control what issues are seen as important.

(Interviewee)

It was also mentioned that if people have a fever they automatically say it is malaria. Some even questioned if trypanosomiasis really exists in humans. ‘If you asked urban people…they would not know about it’ (Interviewee). This may be affecting the narrative that HAT is not important. The disease is neglected because people are ignorant about its effects.

An alternative view is held by those coming from the protection narrative. They believe the disease is protecting undisturbed natural areas from being invaded by people and livestock, and they support the policy decisions based on inaction around eradicating the disease. They cited the effects of deforestation for charcoal, poaching and killing of wildlife, and destruction of the natural environment as areas that were being protected by a lack of funding for trypanosomiasis.

Everyone seemed to agree that trypanosomiasis control used to be higher on the agenda, better funded and the subject of more programmes. Most people looked to other countries, and felt these had a better model for dealing with the disease. There is currently a tendency for responses to be reactive to crises. The Tsetse Section felt that trypanosomiasis is regarded as, 'a forgotten disease because of the way it manifests. It takes a long time to see any effects, whereas other diseases you see [effects] straight away. Trypanosomiasis is chronic. Therefore it has not gotten the attention it deserves.' (Interviewee)

During the interviews this was confirmed by those working for international organisations. They emphasised that should there be a disease outbreak, extra funding is available to assist. As each new disease threat emerges, prior threats are forgotten.

### Poverty alleviation

As shown previously in this paper, trypanosomiasis control is believed to be an important contributor to poverty alleviation by those coming from the poverty narrative. Trypanosomiasis has a disproportionate impact on poor people.

When the fly spreads it stops areas being productive. People have to move away into marginal and unproductive areas. This means that poverty increases and livestock numbers go down. This is a vicious circle and poverty will go up if people can’t own livestock. Livestock is very critical for livelihoods. Without them farmers will be poverty stricken and dependent on the Government. They need help and are dependent on food relief. Fertile areas become no-go areas because of trypanosomiasis. If we don’t deal with this problem we will depend on donors to feed our citizens.

(Interviewee)

Those coming from the poverty narrative believe in moving people on to tsetse-free areas to utilise the land and keep them tsetse free.

Others pointed to further issues outside this debate within Zambia which affect poverty alleviation more. These come from the wider health narrative, and a belief in connectivity in the same way as One Health, and the need for multidisciplinarity and sectors working together.

We could become prosperous if we could sell beef. People could help themselves. There is unrealised potential of beef and livestock. However, there is no motivation to want to move on from traditional herd management to record keeping, dipping, vaccinating, cross breeding […] there is no one to change them to the modern way of thinking. Disease cannot be stopped by being bought. It knows no corruption.

(Interviewee)

The Cotton Association of Zambia pointed to services that the land could provide to help improve poverty levels, but blamed big industry and the international system for this not being possible. They pointed to a belief in a fundamental need for change, not something that can be solved on a project-by-project basis. 'Multinational companies are powerful and we depend on their honesty and transparency. Farmers are not happy with the prices. There is volatility of prices and farmers can’t understand why they are different one year to the next.' (Interviewee)

Examples were given of how disease affects cotton farming, and of the interconnectivity between livestock, farming, health and poverty.

When livestock disease strikes, a lot of farmers lose out and hectares of cotton grown is reduced because farmers do not have cattle to till the land. If you have more cattle it is easier in terms of labour. If you don’t have them then you have to rely on tractors which are expensive and difficult to maintain. If an animal falls sick from anything then you get a lower cotton yield and are forced to reduce the hectares you can grow on.

(Interviewee)

The link with other diseases was also made clear. For example, 'HIV/AIDS is an important health issue. If the husband is sick then the woman can’t go and work in the fields as she has to look after him. If the woman gets sick and dies then you lose the cotton producer. So both is bad. If you lose a woman to any illness then you lose a cotton farmer.' (Interviewee). Thus the livelihoods and poverty levels of households are affected.

Those from the protection narrative believe that eradicating the tsetse fly is not key to poverty alleviation. Instead, people and animals should be kept away from uninhabited land, which should be protected and left to wildlife. Some coming from this perspective have financial gain in mind, for example, from hunting and tourism, while others are environmentalists. In reality, the link between humans, animals and the environment is key, as the welfare of each is interrelated with the others. In many areas, the population depends on wildlife as a source of their livelihood, particularly in the form of both legal and illegal hunting. In the 1940s wildlife ownership changed from traditional to state control [7]. Now, in both GMAs and national parks wildlife are constitutionally the property of the state, and hunting in GMAs requires expensive licenses, restricting traditional hunting. There is some community based natural resource management and some hunting income goes to local communities via community resource boards. However, denial of legal access to protein resources causes negative sentiments among local residents towards Government wildlife policies and has not helped the poverty levels in the area [25]. Related to this issue is that, as a result of hunting, many wild animals retreat to the national parks, contributing to the high density of the tsetse population there.

### Environmental considerations and conservation of biodiversity

In Zambia, human encroachment of protected areas is more prevalent than in most African countries, and ‘2,500–3,000 km^2^ of land are deforested annually’ [9]. Deforestation because of charcoal production, clearing land for farming and livestock keeping, burning for wild honey collection, and hunting are contributing to loss of habitat for wildlife and tsetse [26]. When trees are cut down people plant cotton and conduct other livelihood activities, which have a resulting impact on tsetse, human and livestock populations, thereby affecting disease levels. Every interviewee stated that the biggest ecosystem issue is deforestation and that there was a need for, ‘socially sustainable charcoal[…]and reforestation’ (Interviewee), which is increasing. Currently, it is exported to neighbouring countries, as well as urban Zambia, yet Zambia imports from South Africa. ‘Zambia has one of the highest deforestation rates in the world and this is driven by charcoal' (Interviewee). It is an urban fuel. 'Urban dwellers are driving deforestation […] people in urban areas are not connected to electricity so they use charcoal. The end users get the benefit but the producers make very little profit. There is not much economic benefit for them.' (Interviewee)

Further land-use changes have an impact, include mining which brings roads and new people.

For example, 3,000 people suddenly arrive and create a village with no clinics or services. These must be built. This will happen in lower Zambezi. If you have a project, then people are going to start a township and come with their families and start cultivating. This means that you’ll never be able to get rid of them from the area afterwards. It fundamentally changes the landscape and mines affect local hydrology.

(Interviewee)

There was a belief amongst interviewees that the Government overrode the Environmental Council’s decisions and cared more about disease control than environmental issues.

Another complex element of the ecosystem is wildlife which may be an ecosystem service, but can also be a disservice. Hunters and those in the wildlife industry pointed out the benefits of wildlife in terms of hunting. They were looking from a protection narrative and complained that, ‘local people believe that the animals belong to the country, so they can use them. They believe that no one can own the animals’ (Interviewee). However, in terms of government policy, as stated, the ownership of wildlife changed from traditional to state control in the 1940s. This is not well regulated and:

Police often benefit from poaching so they don’t prosecute people. There’s corruption, and even though the law provides good punishment it is very seldom applied[…]there’s still lots of poaching and so there’s constant problems.

(Interviewee)

This has an impact, ‘as poaching is so severe the Government buy and move animals from game farms to the national parks. This is very random wildlife management and not well thought through. There are no effective policies.’ (Interviewee)

However, when we conducted some participatory workshops with local people in the Eastern Province, there existed what the villagers described as an effective anti-poaching system. Villagers said, that although there were a few areas with poaching issues, they do not hunt in the GMAs and one of the women in the village used to be a scout to try and catch those hunting there. She used to camp up to ten nights at a time and if the group she was with heard guns they would chase the poachers. It was mentioned what has been pointed out in other research, which is that, due to competition for space and resources, there is increasing interaction between people and wildlife which increases human-wildlife conflicts. Crop damage by wildlife has a harmful effect on food production in rural areas and damages people’s ability to have a secure livelihood [27,28]. This loss of crops often leads to negative perceptions of wildlife among farmers which is a key social element of human-wildlife conflicts, potentially leading to retaliatory killings. The complex interplay between policies protecting wildlife and the environment, providing land for people and controlling disease, has led to close contact with wildlife and tsetse during many socioeconomic activities for the local people in the area, predisposing individuals to HAT. This is an issue not just for hunting, but also for other activities such as herding, fishing, collection of firewood, producing charcoal and crop farming. Gender roles are often clearly defined and men and women have different roles such as caring for livestock, farming and gathering (Rutto et al 2013). Culture determines this gender-specific and these differences in daily activities ‘could alter interactions between hosts, parasites and vectors, thus impacting on vector-borne diseases’ [29].

## Discussion

### Competing narratives

Each of the narratives described in this paper have different perspectives on disease, development and the relative importance of environmental, health related, poverty related and economically influenced issues in affecting control options and, therefore, disease incidence. These compete in an environment where data on incidence and impact are uncertain, funding is limited and there are debates among the scientific community about the best course of action. Decision makers are influenced by the fashions of donors, scientific debates and the political environment. Recognising and understanding different perspectives is important and can contribute to the understanding of trypanosomiasis control decisions and their impact on disease, poverty and the environment.

### Potential benefits from One Health

It is clear from interviews and research that there are complex interacting factors affecting trypanosomiasis, supporting the need for joined-up working. This system of interrelated environmental, human and animal health components, each operating in ways that are highly dynamic and uncertain, is exemplified in this Zambian case study. The different fly vectors are highly dependent on particular habitats for their survival and so ecological and land-use change has a major impact on fly populations, and the associated disease risks [30]. This study illustrates how disease and poverty can be intricately connected with environmental issues and ecosystem services. Due to the political structure in Zambia the use of ecosystem services is not regulated. Increasing population growth, along with internal migration to new areas, has led to increased pressure on the provision of ecosystem services, with resulting effects on the environment, disease and livelihoods. Government motivation for eradicating tsetse is influenced by population growth and the need for more areas for settlement and for cattle.

This knowledge has led to the conclusion that trypanosomiasis in Zambia could be dealt with very effectively using a One Health approach, with increased interdisciplinary collaborations and communications in all aspects of zoonotic disease management and policy involving those working for the benefit of people, animals and the environment. This approach may be able to offer potential solutions through cross-sector working, in particular, the effects of policies by different government departments can be assessed for their impact on disease, so they can be managed to work in synergy. Current collaborations could be formalised to ensure that management of trypanosomiasis and its effects reflected a One Health perspective. Especially in the context of reduced trypanosomiasis funding, a this approach can be of great importance to the health of populations as it can increase understanding and the economic benefits of sharing of resources. Additionally, as trypanosomiasis disproportionately affects the poor, poverty reduction must be integrated into policies and strategies. Too often, disease control impacts on people’s livelihoods and other aspects of their lives are not assessed, and these may be greater than the costs of the disease. A One Health approach must be justice-based and incorporate a balanced assessment of the pros and cons of disease control.

While One Health is ‘theoretically and economically attractive’, a significant change in the political arena and policy including increased state capacity is needed if it is to be realised. Currently there are financial and institutional obstacles stopping this approach from being implemented, particularly in developing countries such as Zambia, where there are many health and development issues needing attention and investment [31]. Broad institutional changes, capacity building and dissemination of information forming the evidence base on trypanosomiasis are required as well as inter-ministerial platforms to coordinate policy and action for zoonoses control. Cross-sector working can ensure improved preparedness and contingency planning, cost-sharing between sectors, increased equity in health, and increased sharing of logistics and service provision costs [32]. Representations from different sectors in a One Health department, or similar forum which has overarching control over decisions related to land management in the human-wildlife interface could be useful, but there are difficulties involved in enacting this. However, without such a structure different departments could continue in a power struggle, where those with ultimate power over different areas continue to act the way they feel is best, without considering the One Health approach. Additionally, there must be added value to working together and increased benefits for all sectors, and with appropriate management as suggested, this is possible.

Monitoring needs to shift from a focus on a disease to whole-system surveillance, looking at interactions between disease drivers, disease incidence at the community level, and poverty and equity impacts. A stronger evidence base could lead stakeholders to reassess their perspective and views on trypanosomiasis control. Although, even with this information, disease emergence is inherently uncertain and requires an approach centred on adaptive management, combining surveillance and careful experimentation. This better monitoring should allow increased understanding of the impact of policy options and greater motivation for control, as well as allowing narratives to be based on a solid evidence base [33].

To be able to achieve this, new organisational arrangement, diverse expertise and the direct involvement of local people affected are needed. The interviews conducted showed that the debate is centred on certain professional and bureaucratic interests, and that those living with the disease in poor and marginal areas have little or no say in what priorities are decided upon. Most mention of local people was centred around complaints about their impact on wildlife and the environment. However, assessing their interests is important as their support can be extremely beneficial in ensuring that activities become embedded in communities and continue after projects and funding have ended. Local support also ensures that a sufficient amount of people contribute towards creating public goods [34, 35]. This assessment could be achieved through participatory approaches, especially in areas where detailed data sources are often unavailable. Until now, little attention has been paid to the gains that can be made from community participation as most trypanosomiasis projects are ‘designed by veterinarians or entomologists with little, if any, input from social scientists’ [36]. Some reluctance stems from fears of local people changing the implementation to fit with local objectives and understanding, away from technical advice and there appears to be little will to address this deficit [36]. Even where there has been some local involvement and projects focusing on creation of local awareness about the issues, this has stopped short of including local communities’ knowledge and expertise as evidence for decision making and involving them in policy decisions. A One Health approach could be a great benefit in breaking down the barriers between social scientists, natural scientists and the expertise of the community. Knowledge on disease control is available and we should not wait for a human epidemic to come before implementing these changes.
